# Pregnancy outcomes of intrauterine insemination in young patients with diminished ovarian reserve: a multicenter cohort study

**DOI:** 10.1186/s40001-023-01377-z

**Published:** 2023-10-05

**Authors:** Min Zhao, Qing Huan, Lisa Huang, Lin Yang, Meng Dong

**Affiliations:** 1 Mianyang Central Hospital, School of Medicine, University of Electronic Science and Technology of China, Mianyang, 621000 Sichuan China; 2 Shandong Key Laboratory of Reproductive Medicine, Department of Obstetrics and Gynecology, Shandong Provincial Hospital Affiliated to Shandong First Medical University, Jinan, 250021 Shandong China; 3 Center of Reproductive Medicine, Shengjing Hospital of China Medical University, Shenyang, 110072 Liaoning China

**Keywords:** Diminished ovarian reserve, Pregnancy outcome, Intrauterine insemination, Young, Live birth, Miscarriage

## Abstract

**Background:**

To date, there is no consensus on whether intrauterine insemination (IUI) treatment is required in young patients with diminished ovarian reserve (DOR). Pregnancy outcomes in young DOR patients undergoing IUI are controversial. The existing studies are all single-center studies, with no existing multicenter cohort studies. The purpose of this multicenter study was to investigate the pregnancy outcomes of young DOR patients undergoing IUI.

**Methods:**

This multicenter cohort study included a total of 4600 cycles in 2204 infertile patients who underwent IUI treatment in three reproductive medical centers between September 2018 and January 2022. The research subjects were divided into two groups according to their serum anti-Müllerian hormone (AMH) levels. Propensity score matching (PSM) was used to match the research subjects at a ratio of 1:4. The pregnancy outcomes in the two groups were compared.

**Results:**

There was no significant difference in the clinical pregnancy rates (CPR), biochemical rates, and ectopic pregnancy rates between the two groups (*P* > 0.05). There were, however, significant differences in the miscarriage rates between the groups (*P* < 0.05). The live birth rates (LBR) were 6.6 vs. 9.9 between the two groups. The multivariable logistic regression models reveal that body mass index, AMH were significantly correlated with CPR; AMH were significantly correlated with LBR; BMI, follicle stimulating hormone were significantly correlated with miscarriage rate.

**Conclusions:**

The clinical pregnancy rate of DOR patients was not significantly different from that of NOR patients; however, the miscarriage rates were significantly different from those of NOR patients.

**Supplementary Information:**

The online version contains supplementary material available at 10.1186/s40001-023-01377-z.

## Introduction

With age, oocyte and follicle pools gradually decrease, which is accompanied by a decline in female fertility [1]. However, women of the same age may have different fertility rates owing to ovarian responses. Ovarian reserve (OR) refers to the ability of follicles in the cortical region of the ovary to grow, develop, and form fertilizable oocytes, which is dependent on the number and quality of follicles in the ovary [2]. Diminished ovarian reserve refers to the reduced ability of the ovaries to produce oocytes due to advanced age and congenital, medical, or surgical reasons. OR is often assessed clinically using multiple tests, including blood biomarkers and ultrasound [2, 3]. In all experiments assessing OR, the serum anti-Müllerian hormone (AMH) concentration and antral follicle count (AFC) were considered to be indicators of the optimal value for evaluating ovarian function [2, 3]. An experimental model further confirmed that AMH and AFC can accurately predict the size of the antral follicle pool and ovarian response to gonadotropin stimulation [4, 5].

AMH and AFC are important indicators for diagnosing DOR [6, 7]. DOR describes women of reproductive age with regular menses whose response to ovarian stimulation or fecundity is reduced compared to that in women of the same age [2, 5]. However, whether or not young patients with DOR require IUI treatment has not yet been clarified. IUI is a common first-line treatment for infertility in women. Patients with low fertility due to mild male factors, unknown causes, or endometriosis are usually treated with IUI before in vitro fertilization-embryo transfer (IVF-ET) treatment [8, 9]. However, there is controversy regarding whether young infertile women with DOR are preferably treated with IUI. A study examining DOR patients aged < 35 years, compared with age-matched patients with normal ovarian reserve, compared 370 DOR patients with AMH < 1.0 ng/mL to 2649 patients with AMH ≥ 1.0 ng/ml. There were no significant differences in the HCG-positive, biochemical loss, clinical pregnancy, first pregnancy, and live birth rates between the two groups [10]. In another study, which grouped patients according to the Poseidon criteria (75 DOR patients with 153 cycles and 287 NOR patients with 617 cycles), pregnancy, miscarriage, and multiple pregnancy rates were similar among the groups [11]. A study in Chinese Taipei obtained the opposite result; their study concluded that AMH levels had a significant independent effect on pregnancy outcomes, and low AMH levels were associated with a lower chance of clinical pregnancy [12].

To date, there is no consensus on whether IUI is required in young patients with DOR. Pregnancy outcomes in young DOR patients compared to those in NOR patients undergoing IUI are controversial. The existing studies are all single-center studies, with no existing multicenter cohort studies. This study included data from three reproductive centers: one in northeastern China, one in southwestern China, and one in eastern China. The purpose of this multicenter study was to investigate the pregnancy outcomes of young (aged < 35 years) DOR patients undergoing IUI. We aim to explore the clinical application value of IUI in young patients with DOR. Through this research, we will explore the best fertility treatment options for young patients with DOR and provide reference data for clinical decision-making.

## Materials and methods

### Participants

This multicenter cohort study included a total of 4600 cycles in 2204 infertile patients who underwent IUI treatment in three reproductive medical centers (Mianyang Central Hospital, affiliated to University of Electronic Science and Technology of China; Shengjing Hospital, affiliated to China Medical University; and Shandong Provincial Hospital, affiliated to Shandong First Medical University) between September 2018 and January 2022 (Additional file 1: Fig. S1). All patients were artificially inseminated. The inclusion criteria were as follows: aged ≤ 35 years, AMH < 1.1 ng/ml. The exclusion criteria included endocrine system diseases, abnormal thyroid function, hyperprolactinemia, endometriosis, uterine malformation, intrauterine adhesions, urinary system infection, combined hypertension and diabetes, history of tuberculosis, and total number of spermatozoa with forward motility after semen optimization on IUI day < 10 × 10^6^. This study has been approved by the ethics committee (approval number: S-2021-013) and conforms to the principles of the Declaration of Helsinki.

### Grouping criteria

According to the Bologna consensus and Poseidon criteria [6, 7], the research subjects were divided into two groups according to their serum AMH levels (experimental group, < 1.1 ng/ml, and control group, ≥ 1.1 ng/ml). Propensity score matching (PSM) was used to match the research subjects at a ratio of 1:4.

### Ovulation protocols

Ovulation stimulation protocols included natural, microstimulated (clomiphene and letrozole), and gonadotropin-stimulated (Gn) cycles. In the natural cycle, vaginal B-ultrasound was used to monitor the development of follicles on the 10–12th day of menstruation. The microstimulated ovulation induction cycle had two ways, clomiphene and letrozole. Clomiphene (100 mg/day) or letrozole (5 mg/day) was administered from days 2–4 of the menstrual cycle for 5 days. In the Gn cycle, gonadotropin treatment began on days 2–4 of the menstrual cycle to stimulate the cycle.

### Insemination timing and semen collection

When the diameter of one or two dominant follicles was ≥ 18 mm, or when there was a peak of luteinizing hormone in the urine or blood, a chorionic gonadotropin (HCG) 5000–10,000 U trigger was given. IUI was performed after 24–36 h. If three or more dominant follicles developed, the cycle was canceled and not included in this study. On the day of insemination, semen samples were collected and processed according to standard procedures specified by the WHO. Gradient centrifugation was used to process semen after masturbation. After carefully checking the patient's information, an IUI tube was used to aspirate the sperm suspension (0.5 mL), the IUI tube was gently inserted into the uterine cavity, and the sperm suspension was slowly injected. After the operation, the patient was placed in the supine position for 30 min to observe whether there was any special discomfort.

### Luteal support and follow-up

Postoperative routine luteal support included oral dydrogesterone (10 mg, bid) for 14 days after ovulation. All patients underwent a blood HCG test 14 days postoperatively. For those with positive blood HCG (> 5 IU), a vaginal B-ultrasound examination was performed 25–40 days after IUI. Clinical pregnancy was confirmed if vaginal ultrasonography detected an intrauterine gestational sac and the fetal heartbeat. Miscarriage was defined as the detection of an intrauterine gestational sac on ultrasonography, but the pregnancy did not progress or was spontaneously lost. Live births were defined as neonates delivered after 28 weeks with signs of life within 7 days. The biochemical loss was defined as a positive serum HCG followed by a spontaneous decline in the absence of intrauterine pregnancy on ultrasound. Ectopic pregnancy is defined as the extrauterine gestational sac that was visualized on transvaginal ultrasound.

### Observation measures

The main research index was to compare the clinical pregnancy rates (CPR) of the two groups, and the secondary research indices were biochemical loss, miscarriage, ectopic pregnancy, and live birth rates (LBR). The CPR was calculated as follows: CPR = number of pregnant mothers with clinical pregnancy/women enrolled in the corresponding group [13]. LBR = number of pregnant mothers with live birth/women enrolled in the corresponding group. Miscarriage rate = number of pregnant mothers with miscarriage pregnancy/women enrolled in the corresponding group.

### Statistical analysis

SPSS software (version 23.0) was used for the data analysis. The categorical variables are summarized as counts and percentages (*n*, %), and the comparison between groups was performed using the Chi-square test. The continuous variables are expressed as mean ± standard deviation (mean ± SD), and the corrected *t*-test was used to compare these data. PSM was performed to balance baseline characteristics between the two groups at a ratio of 1:4. The factors influencing the pregnancy outcomes were analyzed using multivariate regression. All tests were two-tailed, and a *P*-value < 0.05 indicated statistical significance.

## Results

### Characteristics of patients

In total, 2204 patients with 4600 clinical cycles were included in this study. Before matching, the experimental and control groups contained 365 and 4235 cycles, respectively. There were significant differences in age, AMH, FSH, luteinizing hormone (LH), testosterone (T), infertility diagnosis, AFC, and controlled ovarian stimulation (COS) protocol between the two groups (*P* < 0.05) (Table 1, Additional file 2: Fig. S2). After matching, the experimental and control groups contained 365 and 1440 cycles, respectively. After PSM matching, there were significant differences in FSH, LH, infertility diagnosis, AFC, IUI day endometrial thickness, and COS protocol between the two groups. However, there were no significant differences in age, infertility duration, BMI, T, or sperm count (*P* > 0.05) (Table 1, Additional file 2: Fig. S2).

**Table 1:** Demographic characteristics of the study population before matching (*n* = 4600)

	Before matching			After matching		
	DOR (*n* = 365)	NOR (*n* = 4235)	*P*-value	DOR (*n* = 365)	NOR (*n* = 1440)	*P*-value
	Mean ± SD/*n* (%)	Mean ± SD/*n* (%)		Mean ± SD/*n* (%)	Mean ± SD/*n* (%)	
Female age (years)	30.93 ± 2.89	29.89 ± 3.05	< 0.01^**^	30.93 ± 2.89	30.88 ± 2.80	0.73
Male age (years)	33.05 ± 4.04	31.67 ± 3.71	< 0.01^**^	33.05 ± 4.04	32.85 ± 3.86	0.37
BMI (kg/m^2^)	22.60 ± 3.80	22.86 ± 3.95	0.24	22.60 ± 3.80	22.61 ± 3.73	0.98
Infertility duration	3.29 ± 2.12	3.25 ± 2.02	0.74	3.29 ± 2.12	3.44 ± 2.16	0.24
Sex hormone levels						
AMH (ng/ml)	0.77 ± 0.27	5.45 ± 3.77	< 0.01^**^	0.77 ± 0.27	5.17 ± 3.53	< 0.01^**^
FSH (IU/L)	7.93 ± 3.68	6.39 ± 3.23	< 0.01^**^	7.93 ± 3.68	6.52 ± 3.83	< 0.01^**^
LH (IU/L)	4.71 ± 8.29	5.66 ± 5.33	0.03^*^	4.71 ± 8.29	5.69 ± 5.63	< 0.01^**^
*E*_2_ (pmol/L)	47.41 ± 35.35	49.37 ± 51.74	0.45	47.41 ± 35.35	51.52 ± 52.87	0.16
* T* (ng/ml)	0.88 ± 3.19	0.61 ± 2.25	0.04^*^	0.88 ± 3.19	0.64 ± 2.20	1.00
Infertility diagnosis			< 0.01^**^			< 0.01^**^
Uterine	11 (3.0)	893 (21.1)		11 (3.0)	358 (24.9)	
Ovulatory	21 (5.8)	683 (16.1)		21 (5.8)	224 (15.6)	
Endometriosis	9 (2.5)	96 (2.3)		9 (2.5)	33 (2.3)	
Male factor	106 (29.0)	1832 (43.3)		106 (29.0)	561 (39.0)	
Unexplained	24 (6.6)	731 (17.3)		24 (6.6)	264 (18.3)	
Decreased ovarian reserve	194 (53.2)	–0		194 (53.2)	0	
AFC	5.18 ± 5.51	11.80 ± 7.28	< 0.01^**^	5.18 ± 5.51	11.01 ± 6.72	< 0.01^**^
Seminal parameters						
Sperm concentration, *10^6^/ml	66.83 ± 28.86	69.52 ± 31.86	0.12	66.83 ± 28.86	68.85 ± 33.50	0.29
Sperm progressive motility (%)	33.69 ± 18.34	33.66 ± 17.88	0.97	33.69 ± 18.34	33.56 ± 17.41	0.90
Endometrial thickness (mm)	10.30 ± 3.41	10.68 ± 3.64	0.06	10.30 ± 3.41	10.91 ± 3.73	< 0.01^**^
COH protocol			< 0.01^**^			< 0.01^**^
Natural cycle	127 (34.8)	1391 (32.8)		127 (34.8)	499 (34.7)	
Clomiphene	42 (11.5)	841 (19.9)		42 (11.5)	298 (20.7)	
Letrozole	145 (39.7)	1346 (31.8)		145 (39.7)	405 (28.1)	
Gn	51 (14.0)	657 (15.5)		51 (14.0)	238 (16.5)	

SD, standard deviation; BMI, body mass index; AMH, anti-Müllerian hormone; FSH, follicle stimulating hormone; LH, luteinizing hormone; E_2_, estradiol; *T*, testosterone; AFC, antral follicle count; COH, controlled ovarian stimulation

^*^P < 0.05, **P < 0.01

### Comparison of pregnancy outcomes

There was no significant difference in the clinical pregnancy rates, biochemical rates, and ectopic pregnancy rates between the two groups (*P* > 0.05). There were, however, significant differences in the miscarriage rates between the groups (*P* < 0.05). The live birth rates were 6.6 vs. 9.9 between the two groups (*P* = 0.05) (Table 2, Additional file 3: Fig. S3).

**Table 2:** Comparison of pregnancy outcomes of two groups (*n* = 1805)

	DOR (*n* = 365)	NOR (*n* = 1440)	*P*-value
	*n* (%)	*n* (%)	
Clinical pregnancy rates	48 (13.2)	190 (13.2)	0.98
Biochemical rates	179 (49.04)	714 (49.58)	0.85
Miscarriage rates	20 (5.5)	38 (2.6)	< 0.01^**^
Ectopic pregnancy rates	4 (1.1)	10 (0.7)	0.44
Live birth rates	24 (6.6)	142 (9.9)	0.05

^*^^*^P < 0.05

### Multivariate regression analysis of factors affecting the pregnancy outcomes

The multivariable logistic regression models were used to analyze relevant factors that affect the pregnancy outcomes. The results reveal that BMI [0.959 (0.925–0.993), *P* < 0.05], AMH [0.938 (0.899–0.978), *P* < 0.05] were significantly correlated with CPR (Table 3, Additional file 4: Fig. S4). For live birth, AMH [0.925 (0.882–0.970), *P* < 0.05] was significantly correlated with LBR (Table 4, Additional file 5: Fig. S5). However, BMI [0.891 (0.839–0.947), *P* < 0.05], FSH [1.152 (1.004–1.323), *P* < 0.05] were significantly correlated with miscarriage rate (Table 5, Additional file 6: Fig. S6). Other factors had no significant effects on pregnancy outcomes (*P* > 0.05) (Tables 3, 4, 5).

**Table 3:** Multivariate logistic regression analysis for the factors to predict clinical pregnancy

Items	B	*SE*	*z*	Wald χ^2^	*P*	OR	95% CI
Female age	0.027	0.033	0.840	0.706	0.401	1.028	0.964–1.096
Male age	− 0.015	0.022	− 0.693	0.480	0.488	0.985	0.943–1.028
BMI	− 0.042	0.018	− 2.321	5.386	0.020	0.959	0.925–0.993
Infertility duration	0.064	0.036	1.789	3.201	0.074	1.066	0.994–1.143
AMH	− 0.064	0.021	− 2.998	8.989	0.003	0.938	0.899–0.978
FSH	0.030	0.031	0.958	0.917	0.338	1.031	0.969–1.096
LH	0.009	0.016	0.545	0.297	0.586	1.009	0.978–1.040
E2	− 0.000	0.002	− 0.225	0.050	0.822	1.000	0.996–1.003
*T*	− 0.005	0.030	− 0.180	0.032	0.857	0.995	0.938–1.054
Infertility diagnosis	− 0.001	0.046	− 0.018	0.000	0.986	0.999	0.914–1.093
AFC	0.017	0.013	1.357	1.843	0.175	1.018	0.992–1.044
Sperm concentration	0.001	0.002	0.362	0.131	0.717	1.001	0.996–1.005
Sperm progressive motility	− 0.000	0.004	− 0.071	0.005	0.944	1.000	0.992–1.008
Endometrial thickness	− 0.000	0.021	− 0.009	0.000	0.993	1.000	0.960–1.041
COH protocol	− 0.066	0.066	− 1.005	1.011	0.315	0.936	0.822–1.065

BMI, body mass index; AMH, anti-Müllerian hormone; FSH, follicle stimulating hormone; LH, luteinizing hormone; E_2_, estradiol; *T*, testosterone; AFC, antral follicle count; COH, controlled ovarian stimulation

**Table 4:** Multivariate logistic regression analysis for the factors to predict live birth

Items	*B*	*SE*	*z*	Wald χ^2^	*p*	OR	95% CI
Female age	0.059	0.038	1.556	2.422	0.120	1.061	0.985–1.143
Male age	− 0.022	0.026	− 0.853	0.728	0.394	0.978	0.930–1.029
BMI	− 0.007	0.022	− 0.299	0.089	0.765	0.993	0.952–1.037
Infertility duration	0.040	0.042	0.963	0.927	0.336	1.041	0.959–1.129
AMH	− 0.078	0.024	− 3.235	10.464	0.001	0.925	0.882–0.970
FSH	0.008	0.028	0.295	0.087	0.768	1.008	0.954–1.066
LH	0.024	0.020	1.167	1.362	0.243	1.024	0.984–1.066
E2	− 0.003	0.002	− 1.534	2.352	0.125	0.997	0.994–1.001
*T*	0.009	0.040	0.218	0.048	0.827	1.009	0.933–1.090
Infertility diagnosis	0.086	0.055	1.562	2.441	0.118	1.089	0.978–1.213
AFC	0.011	0.014	0.737	0.543	0.461	1.011	0.983–1.040
Sperm concentration	− 0.002	0.003	− 0.853	0.728	0.393	0.998	0.993–1.003
Sperm progressive motility	0.006	0.005	1.284	1.649	0.199	1.006	0.997–1.016
Endometrial thickness	0.005	0.025	0.202	0.041	0.840	1.005	0.958–1.055
COH protocol	− 0.111	0.078	− 1.417	2.009	0.156	0.895	0.768–1.043

BMI, body mass index; AMH, anti-Müllerian hormone; FSH, follicle stimulating hormone; LH, luteinizing hormone; E_2_, estradiol; *T*, testosterone; AFC, antral follicle count; COH, controlled ovarian stimulation

**Table 5:** Multivariate logistic regression analysis for the factors to predict miscarriage

Items	*B*	*SE*	*z*	Wald χ^2^	*p*	OR	95% CI
Female age	− 0.095	0.066	− 1.451	2.104	0.147	0.909	0.799–1.034
Male age	0.018	0.043	0.416	0.173	0.678	1.018	0.935–1.109
BMI	− 0.115	0.031	− 3.734	13.940	0.000	0.891	0.839–0.947
Infertility duration	0.069	0.067	1.030	1.061	0.303	1.071	0.940–1.221
AMH	0.020	0.046	0.428	0.183	0.669	1.020	0.932–1.117
FSH	0.142	0.070	2.017	4.067	0.044	1.152	1.004–1.323
LH	− 0.029	0.024	− 1.220	1.487	0.223	0.971	0.927–1.018
E2	0.006	0.004	1.604	2.573	0.109	1.006	0.999–1.014
*T*	0.051	0.089	0.577	0.332	0.564	1.052	0.885–1.252
Infertility diagnosis	− 0.159	0.088	− 1.810	3.277	0.070	0.853	0.718–1.013
AFC	0.018	0.028	0.654	0.428	0.513	1.018	0.965–1.075
Sperm concentration	0.009	0.005	1.702	2.895	0.089	1.009	0.999–1.020
Sperm progressive motility	− 0.011	0.008	− 1.469	2.159	0.142	0.989	0.974–1.004
Endometrial thickness	− 0.022	0.038	− 0.564	0.318	0.573	0.979	0.908–1.055
COH protocol	− 0.031	0.124	− 0.250	0.062	0.803	0.970	0.761–1.235

BMI, body mass index; AMH, anti-Müllerian hormone; FSH, follicle stimulating hormone; LH, luteinizing hormone; E_2_, estradiol; *T*, testosterone; AFC, antral follicle count; COH, controlled ovarian stimulation

## Discussion

The treatment of patients with DOR has always been a research hotspot in the field of reproduction, and its practice is full of challenges. There is relatively little research on this topic, especially for young women with DOR. To our knowledge, this is the first multicenter cohort study to compare the pregnancy outcomes of DOR patients aged < 35 years with those of NOR patients who received IUI treatment. We aimed to explore the clinical value of IUI in DOR patients aged < 35 years. This study found that the clinical pregnancy rate of DOR patients was not significantly different from that of NOR patients; however, the miscarriage rates were significantly different from those of NOR patients. The multivariate regression model revealed that BMI, AMH, and FSH were the factors affecting pregnancy outcomes.

Of the previous studies, only three compared pregnancy outcomes in young patients with DOR treated with IUI, but all were single-center studies [10–12]. A 2020 study [10] in the United States found that in DOR patients aged < 35 years, clinical pregnancy rates and cumulative pregnancy outcomes per cycle after IUI were similar to those in age-matched NOR patients. Additionally, in IUI cycles with gonadotropins, the dose of FSH must be increased to obtain reproductive outcomes similar to those of NOR patients. The cumulative pregnancy and live birth rates were also similar in younger patients with DOR and those with NOR. These data imply quantitative rather than qualitative distinctions between the groups. This is partly consistent with our study, but this study did not compare the difference in miscarriage rates between the two groups. In our study, the live birth rates were 6.6 vs. 9.9 between the two groups (*P* = 0.05).

Kaleli et al. [11] compared pregnancy outcomes in DOR and NOR patients according to the Poseidon criteria, which combine age and AMH levels. They found no difference in clinical pregnancy rates among the IUI groups. Multivariate logistic regression analysis revealed that infertility duration, sperm count, and follicle number were independent predictors of pregnancy (*P* < 0.05). Neither age nor AMH level predicted pregnancy after IUI, which is inconsistent with our conclusions. This may be due to the different ages of the patients included in the different studies, as their female patients were aged 20–39 years. Additionally, they had a small sample size of 770 cases, with different etiology, and their study was a single-center study.

According to the Bologna consensus and Poseidon criteria [6, 7], our research subjects were divided into two groups according to their serum AMH levels (experimental group, < 1.1 ng/ml, and control group, ≥ 1.1 ng/ml). We opted for AMH instead of AFC as an inclusion criterion primarily due to the following considerations. In clinical practice, the diagnosis of DOR involves the utilization of various methods for ovarian reserve testing in women with regular menstruation, including elevated basal FSH levels, low AMH, low AFC, or less commonly, failure of clomiphene citrate challenge test [6, 14, 15]. A global survey conducted among 796 fertility centers revealed that 51% of them considered AMH as the optimal test for assessing ovarian reserve, while 40% favored AFC and only 6% selected basic FSH [16]. By incorporating these findings, we have adhered to both the Bologna consensus and Poseidon criteria, thus opting for AMH instead of AFC.

In our study, AMH [0.925 (0.882–0.970), *P* < 0.05] was independent and significantly correlated with the live birth rate. AMH is significantly associated with age. A single-center retrospective study analyzing the pregnancy outcomes in 1957 women with DOR found that maternal age and the stage and number of embryos transferred were independent factors affecting the live birth rate [17]. Female age is closely related to oocyte quality and quantity, including cytoskeletal abnormalities, decreased mitochondrial numbers, abnormal spindles, increased aneuploidy, and changes in zona pellucida function [18, 19].

There is considerable evidence that IUI can achieve acceptable success rates in cases with mild-to-moderate male factors, unexplained infertility, and endometriosis [20]. However, its efficacy in women suffering DOR remains controversial. Most women with DOR prefer IVF to avoid missing a critical period maximizing their chances as early as possible. However, for patients aged < 35 years, it is unclear whether simple treatments such as IUI need to be omitted. In a retrospective study comparing pregnancy outcomes in DOR patients with 176 IUI cycles and 639 IVF cycles, there was no significant difference in the pregnancy outcomes. Moreover, IVF did not improve pregnancy outcomes in patients with DOR [21]. As this study did not compare the pregnancy outcomes of IUI with those of IVF in people with DOR, further research is needed on whether they should continue IUI treatment or receive IVF as a next step. The decision-making process is particularly crucial for DOR patients, especially those who have risk factors for miscarriage, such as a history of previous miscarriages.

Reproductive capacity includes not only the ability to conceive, but also the ability to bring the fetus to a viable state [22]; therefore, miscarriage is an important indicator of reproductive capacity [5]. A miscarriage may be caused by a decrease in the oocyte quality, leading to faulty meiosis and embryonic aneuploidy [23, 24]. Our study found that patients with DOR had a higher rate of miscarriage than those with NOR. This may be the result of decreased oocyte quality in patients with DOR. This is consistent with the findings of a high-level meta-analysis published in *Human Reproduction Update* [5] that women with low serum AMH concentrations had an increased risk of miscarriage compared with women with moderate or high AMH levels. After subgroup analysis according to the AFC level, an increased miscarriage rate was also observed in younger women (aged < 35 years). Our study found that BMI and FSH levels were significantly correlated with miscarriage rates. Several studies have confirmed the correlation between BMI and miscarriage [25, 26]. Increased BMI can lead to high miscarriage rates in ART, which is consistent with our observations.

Another recent meta-analysis confirmed the association between DOR and the risk of recurrent pregnancy loss [27]. Although the mechanism is currently poorly studied, clinical evidence has indicated that embryos from DOR patients have a higher incidence of aneuploidy [28, 29]. There are also some possible explanations for the reduced OR in patients with DOR, which may also reflect a systemic clinical condition or past exposure that may independently affect the OR and miscarriage. In addition, common pathogenic lesions may affect the ovary (impairing OR formation or accelerating failure) and uterus (impacting its ability to receive embryos). These possibilities suggest that the association between DOR and miscarriage is not causal, but that the two conditions share a common cause [5].

In 2011, the ESHRE formulated the Bologna consensus on a low ovarian response. One of the criteria included an AFC < 5–7 or serum AMH < 0.5–1.1 μg/L [6]. The Poseidon group believes that the quantitative and qualitative parameters, age, AFC, and AMH, should be combined to formulate clinical treatment strategies [7]. Our study adopted the Bologna criteria, with AMH < 1.1 ug/L as the experimental group inclusion criteria. This was combined with the age stratification of the Poseidon criteria to explore the pregnancy outcomes of young DOR patients receiving IUI. In this study, a multicenter retrospective cohort study and the PSM method were used to improve the evidence strength of the study, control confounding, and prevent bias.

In conclusion, this study investigated the pregnancy outcomes of patients with DOR treated with IUI through a multicenter retrospective cohort study. This study found that the IUI CPR of young DOR patients was not significantly different from that of NOR patients; however, the live birth rate was insignificantly lower than that of NOR patients, and the miscarriage rate was significantly increased. To our knowledge, this is the first multicenter study concerning IUI pregnancy outcomes in young patients with DOR, and this study will provide a valuable reference for the clinical treatment of patients with DOR.

### Supplementary Information

**Additional file 1: Fig. S1.** Location distributions of the three cities in this study.

**Additional file 2: Fig. S2.** Propensity Score Matching.

**Additional file 3: Fig. S3.** Comparison of pregnancy outcomes between the two groups.

**Additional file 4: Fig. S4.** Multivariate regression analysis of factors affecting CPR.

**Additional file 5: Fig. 5.** Multivariate regression analysis of factors affecting the LBR.

**Additional file 6: Fig. 6** Multivariate regression analysis of factors affecting the miscarriage rates.

## Data Availability

The data of this study are available from the corresponding author upon reasonable request.
