# Supplementary figures and images for: Pregnancy outcomes of intrauterine insemination in young patients with diminished ovarian reserve: a multicenter cohort study
Source: Eur J Med Res. 2023 Oct 5;28:402. doi: 10.1186/s40001-023-01377-z (PMC10552364; doi:10.1186/s40001-023-01377-z)

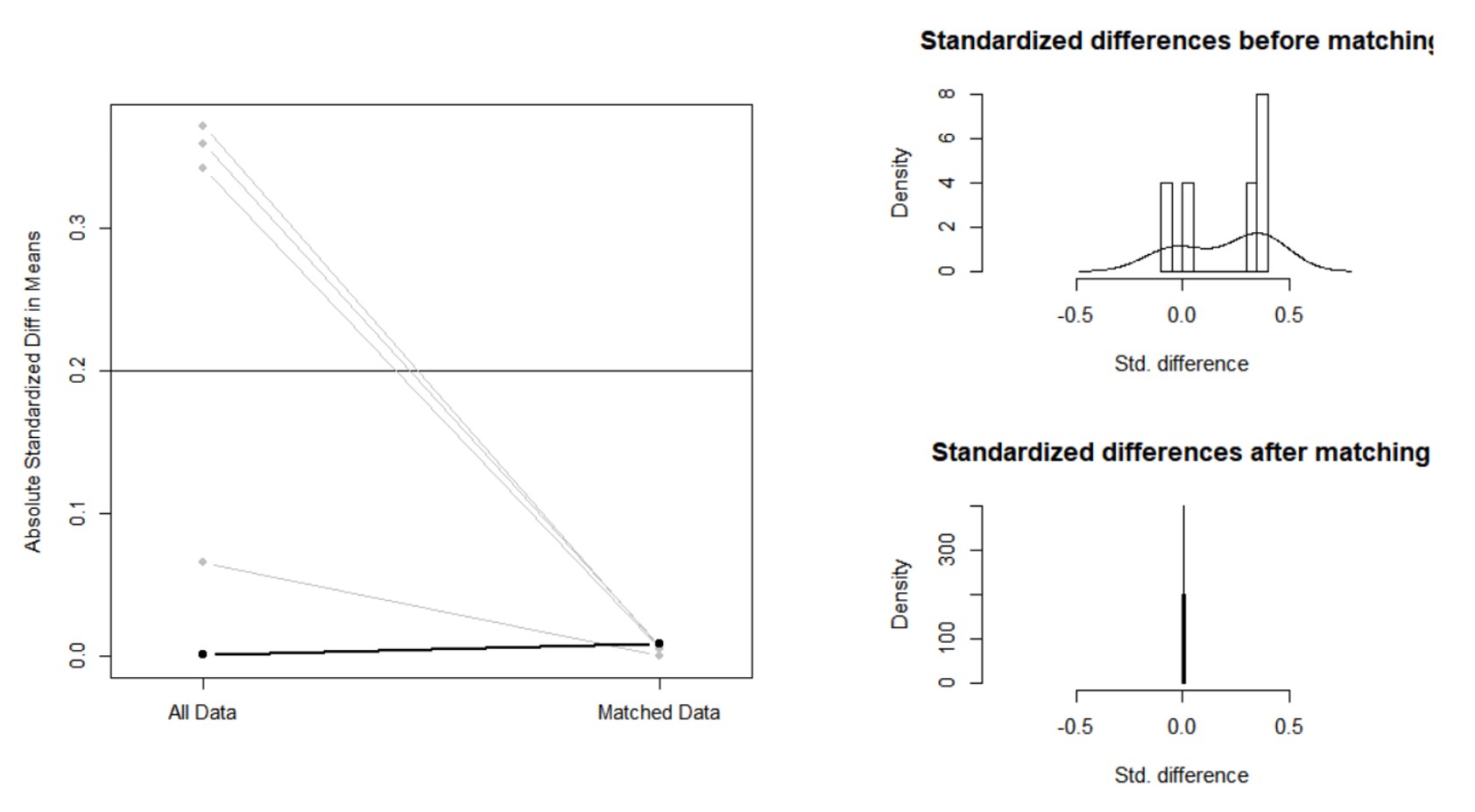

Supplement: Supplementary file 1 — Additional file 1: Fig. S1. Location distributions of the three cities in this study. [file 40001_2023_1377_MOESM1_ESM.jpg]

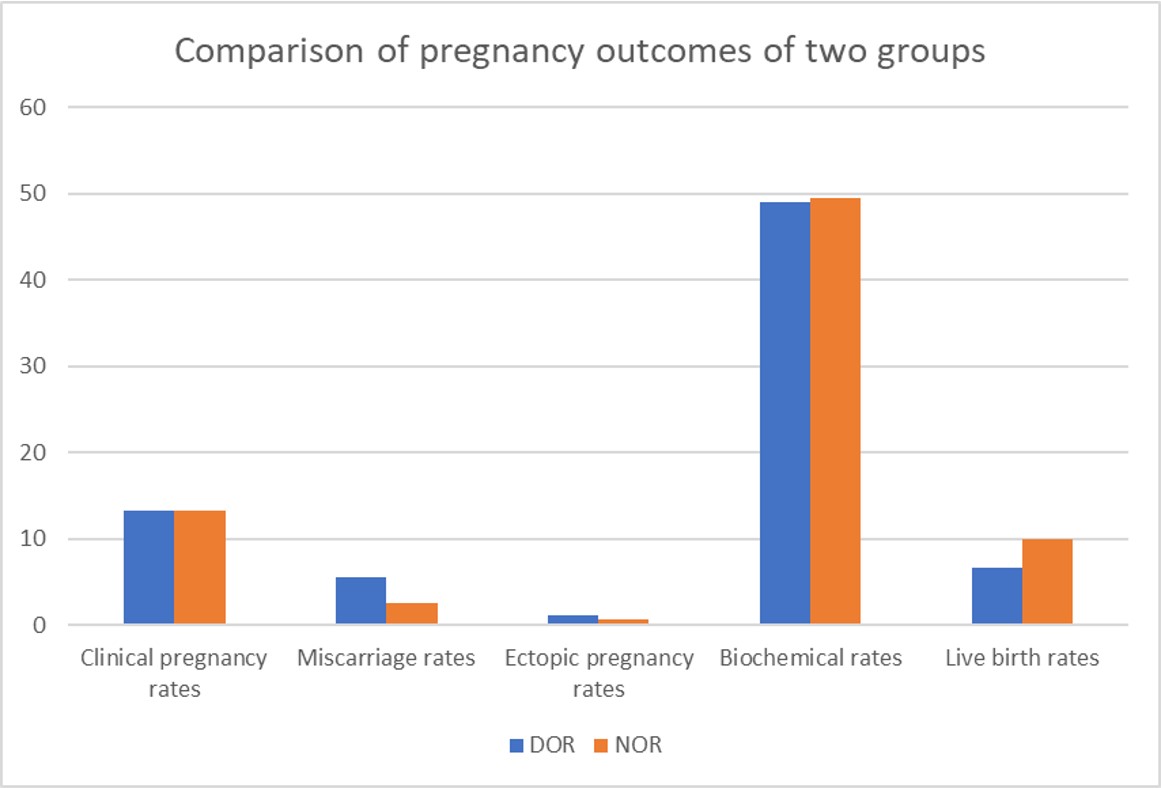

Supplement: Supplementary file 2 — Additional file 2: Fig. S2. Propensity Score Matching. [file 40001_2023_1377_MOESM2_ESM.jpg]

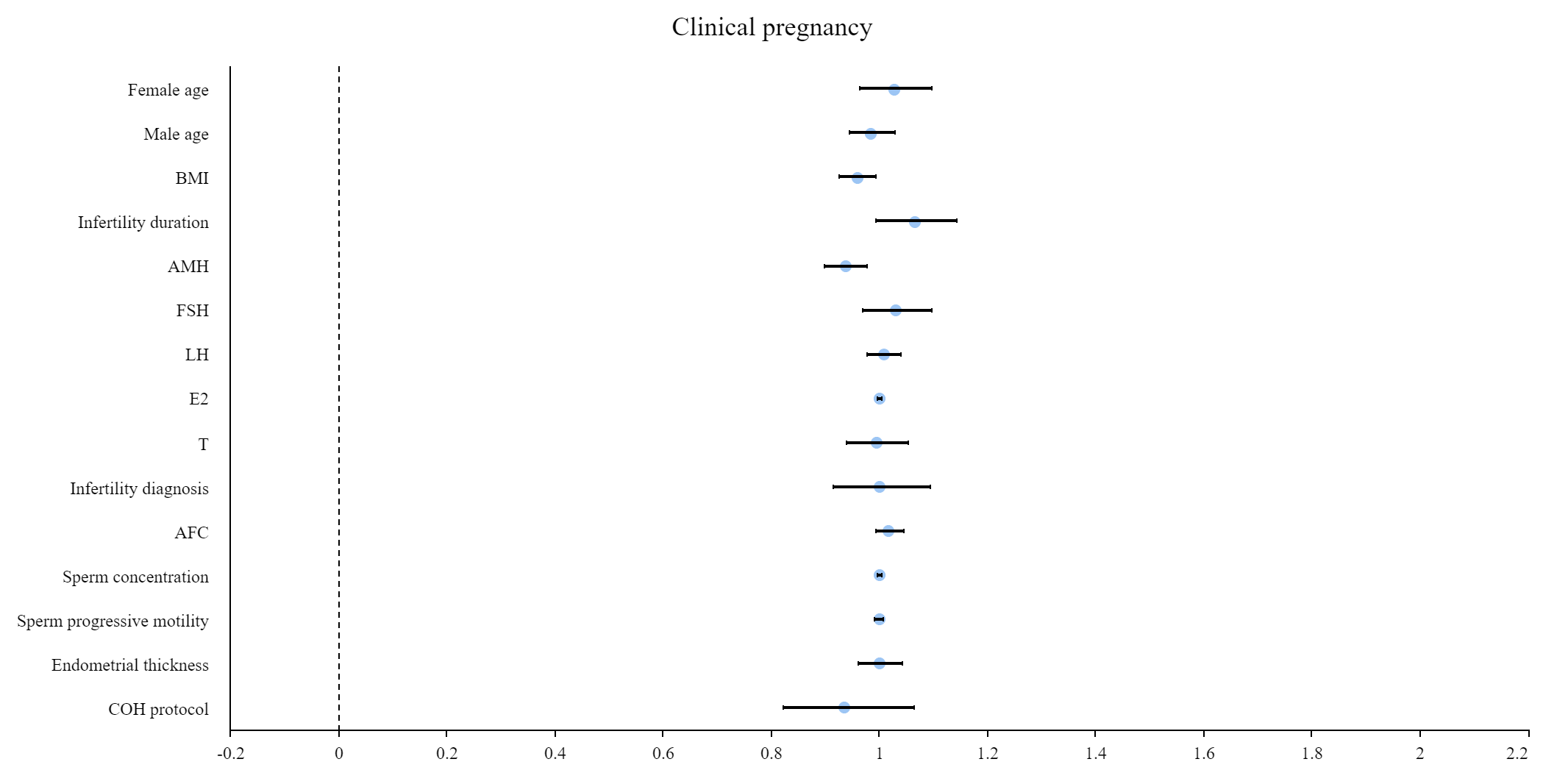

Supplement: Supplementary file 3 — Additional file 3: Fig. S3. Comparison of pregnancy outcomes between the two groups. [file 40001_2023_1377_MOESM3_ESM.jpg]

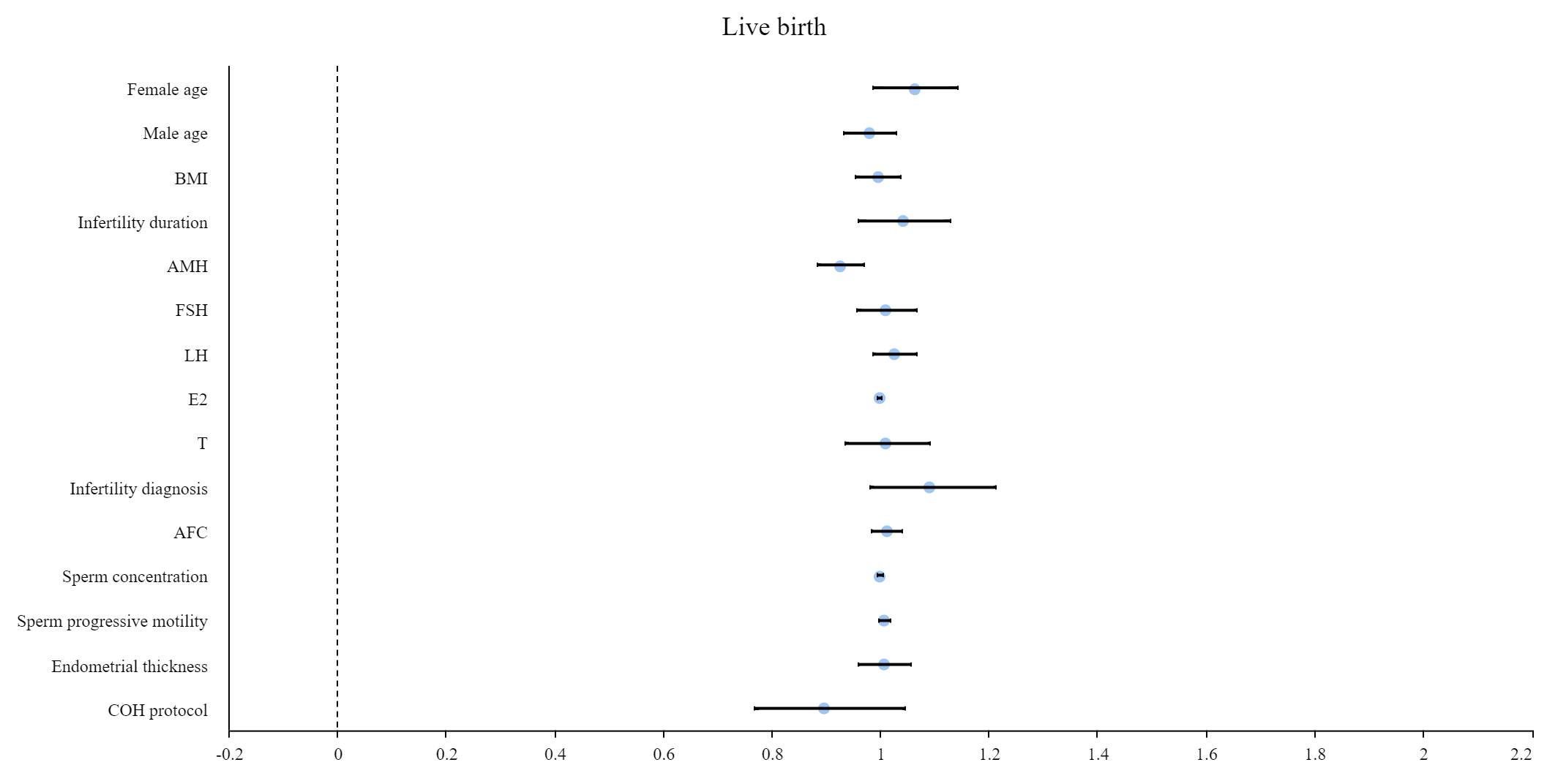

Supplement: Supplementary file 4 — Additional file 4: Fig. S4. Multivariate regression analysis of factors affecting CPR. [file 40001_2023_1377_MOESM4_ESM.jpg]

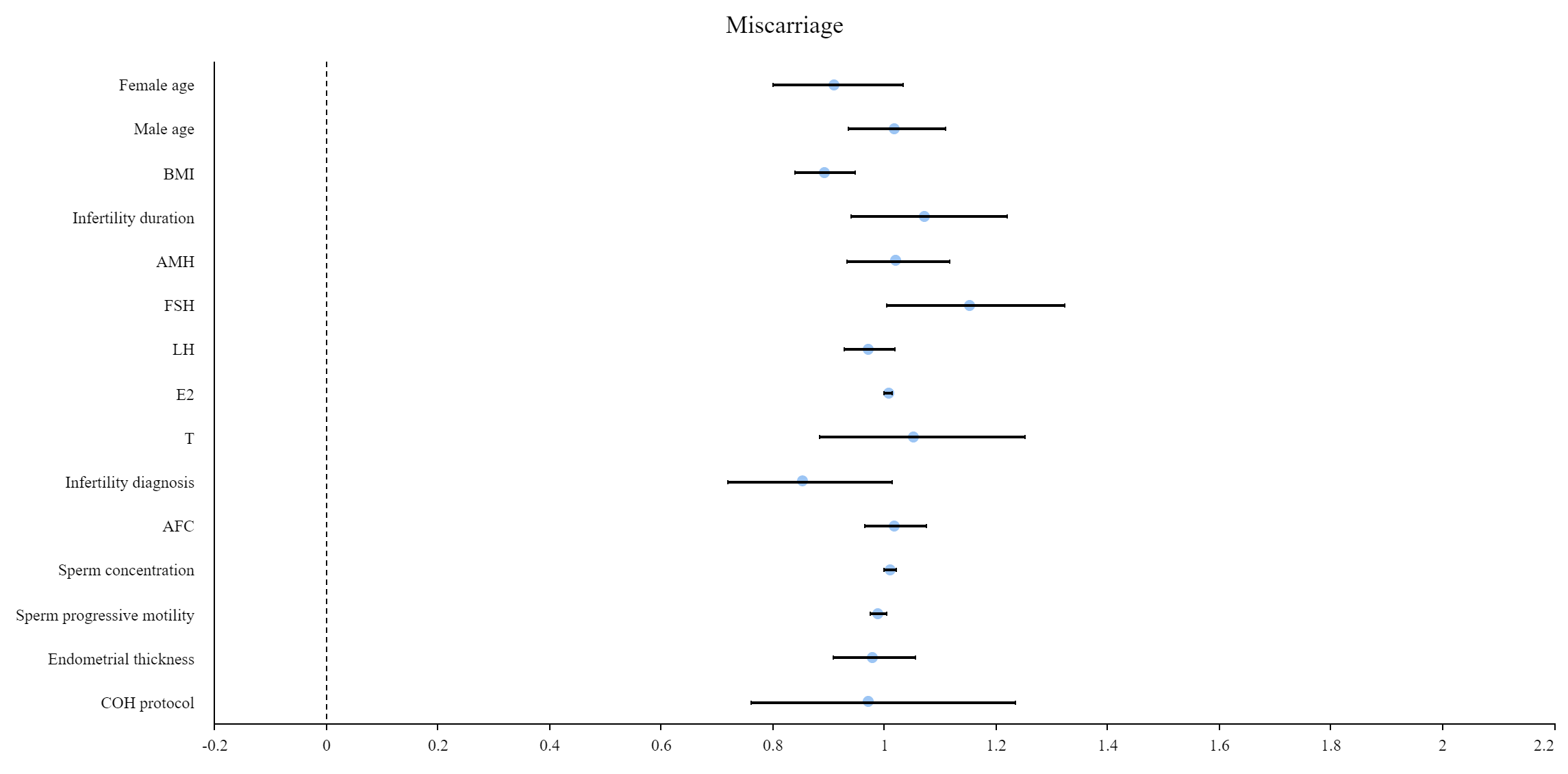

Supplement: Supplementary file 5 — Additional file 5: Fig. 5. Multivariate regression analysis of factors affecting the LBR. [file 40001_2023_1377_MOESM5_ESM.jpg]

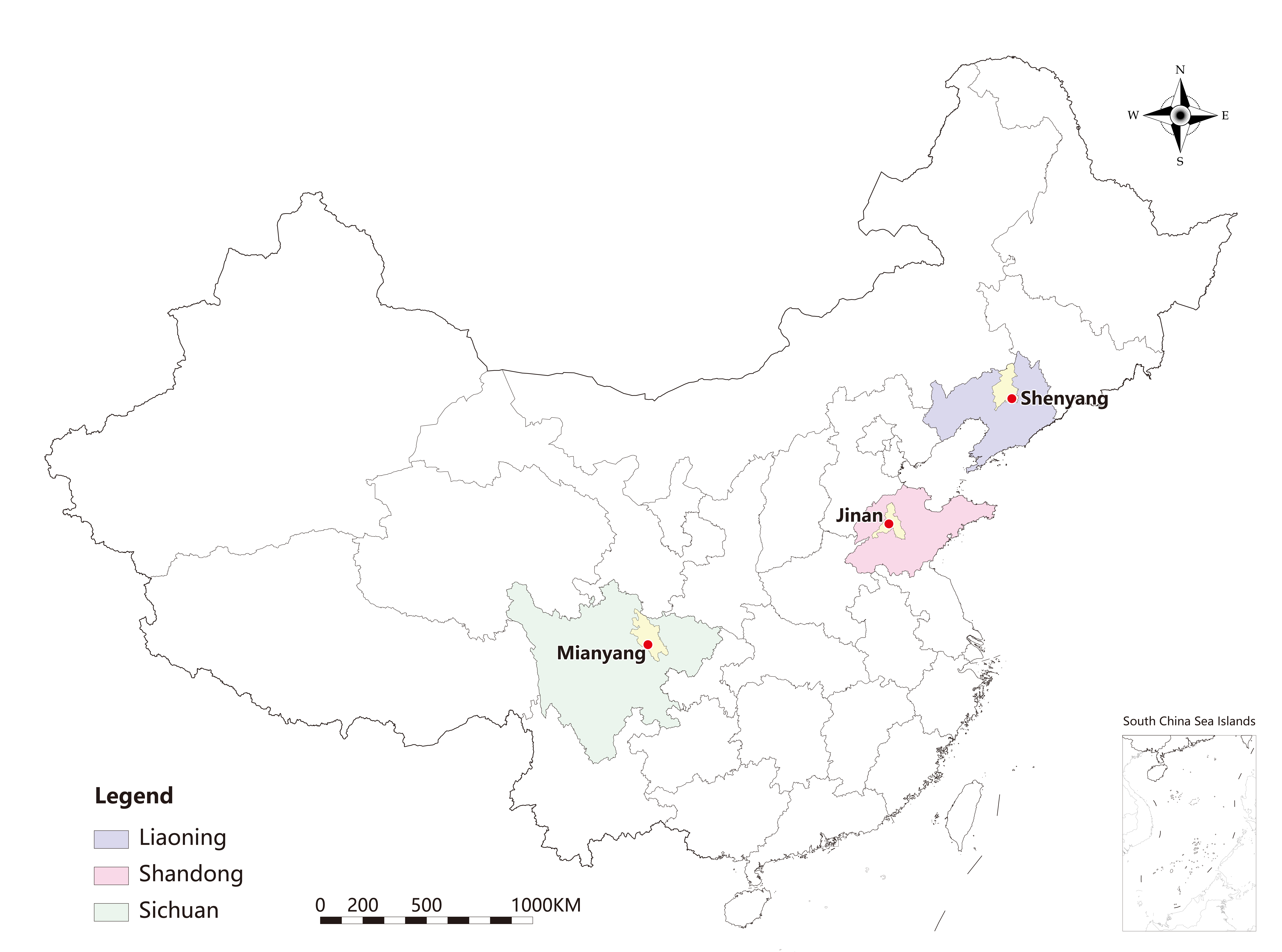

Supplement: Supplementary file 6 — Additional file 6: Fig. 6 Multivariate regression analysis of factors affecting the miscarriage rates. [file 40001_2023_1377_MOESM6_ESM.jpg]
